# Induction of Potent and Durable Neutralizing Antibodies Against SARS-CoV-2 Using a Receptor Binding Domain-Based Immunogen

**DOI:** 10.3389/fimmu.2021.637982

**Published:** 2021-03-11

**Authors:** Vikram Srivastava, Ling Niu, Kruttika S. Phadke, Bryan H. Bellaire, Michael W. Cho

**Affiliations:** ^1^Department of Biomedical Sciences, College of Veterinary Medicine, Iowa State University, Ames, IA, United States; ^2^Department of Veterinary Microbiology and Preventive Medicine, College of Veterinary Medicine, Iowa State University, Ames, IA, United States; ^3^Interdepartmental Microbiology Program, Iowa State University, Ames, IA, United States; ^4^NeoVaxSyn, Inc., Ames, IA, United States

**Keywords:** SARS-CoV-2, COVID-19, vaccine, RBD, neutralizing antibody

## Abstract

A novel betacoronavirus (SARS-CoV-2) that causes severe pneumonia emerged through zoonosis in late 2019. The disease, referred to as COVID-19, has an alarming mortality rate and it is having a devastating effect on the global economy and public health systems. A safe, effective vaccine is urgently needed to halt this pandemic. In this study, immunogenicity of the receptor binding domain (RBD) of spike (S) glycoprotein was examined in mice. Animals were immunized with recombinant RBD antigen intraperitoneally using three different adjuvants (Zn-chitosan, Alhydrogel, and Adju-Phos), and antibody responses were followed for over 5 months. Results showed that potent neutralizing antibodies (nAbs) can be induced with 70% neutralization titer (NT_70_) of ~14,580 against live, infectious viruses. Although antigen-binding antibody titers decreased gradually over time, sufficiently protective levels of nAbs persisted (NT_80_ >2,430) over the 5-month observation period. Results also showed that adjuvants have profound effects on kinetics of nAb induction, total antibody titers, antibody avidity, antibody longevity, and B-cell epitopes targeted by the immune system. In conclusion, a recombinant subunit protein immunogen based on the RBD is a highly promising vaccine candidate. Continued evaluation of RBD immunogenicity using different adjuvants and vaccine regimens could further improve vaccine efficacy.

## Introduction

In late 2019, an outbreak of severe pneumonia (referred to as COVID-19) began in China. The causative pathogen was identified as a novel coronavirus (CoV) designated as SARS-CoV-2 (*a.k.a*. 2019-nCoV). The virus infection resulted in high incidents of fatal pneumonia with clinical symptoms resembling those of SARS-CoV infections during the 2002–03 epidemic. They include persistent fever, chills/rigor, myalgia, malaise, dry cough, headache, and dyspnoea (1). SARS-CoV-2 is believed to have emerged through zoonosis, but the virus is transmitted efficiently from human to human, even prior to onset of symptoms (2), via droplets/aerosol from coughing or sneezing, or direct contact. Consequently, the virus has been able to spread rapidly. In March of 2020, the World Health Organization (WHO) officially declared COVID-19 as a pandemic. As of early December 2020, over 65.4 million confirmed cases with over 1.5 million deaths have been reported in 191 countries (3). The pandemic is having a devastating impact on the global economy and public health systems worldwide. Therefore, a safe and highly protective vaccine is urgently needed.

SARS-CoV-2 belongs to genus *betacoronavirus* of *Coronaviridae* family. Its single strand RNA genome ranges from 29,825 to 29,902 bases long. The virus is most closely related to a bat coronavirus RaTG13 strain (nucleotide sequence identity of ~96%) (4). SARS-CoV-2 is more distantly related to SARS-CoV (82% identity). However, both viruses utilize angiotensin converting enzyme 2 (ACE2) as a receptor (4). Binding of ACE2 and cellular entry is mediated by spike (S) glycoprotein, which is 1,273 amino acids long. S proteins of the two viruses are 76% identical.

**Figure 1:**
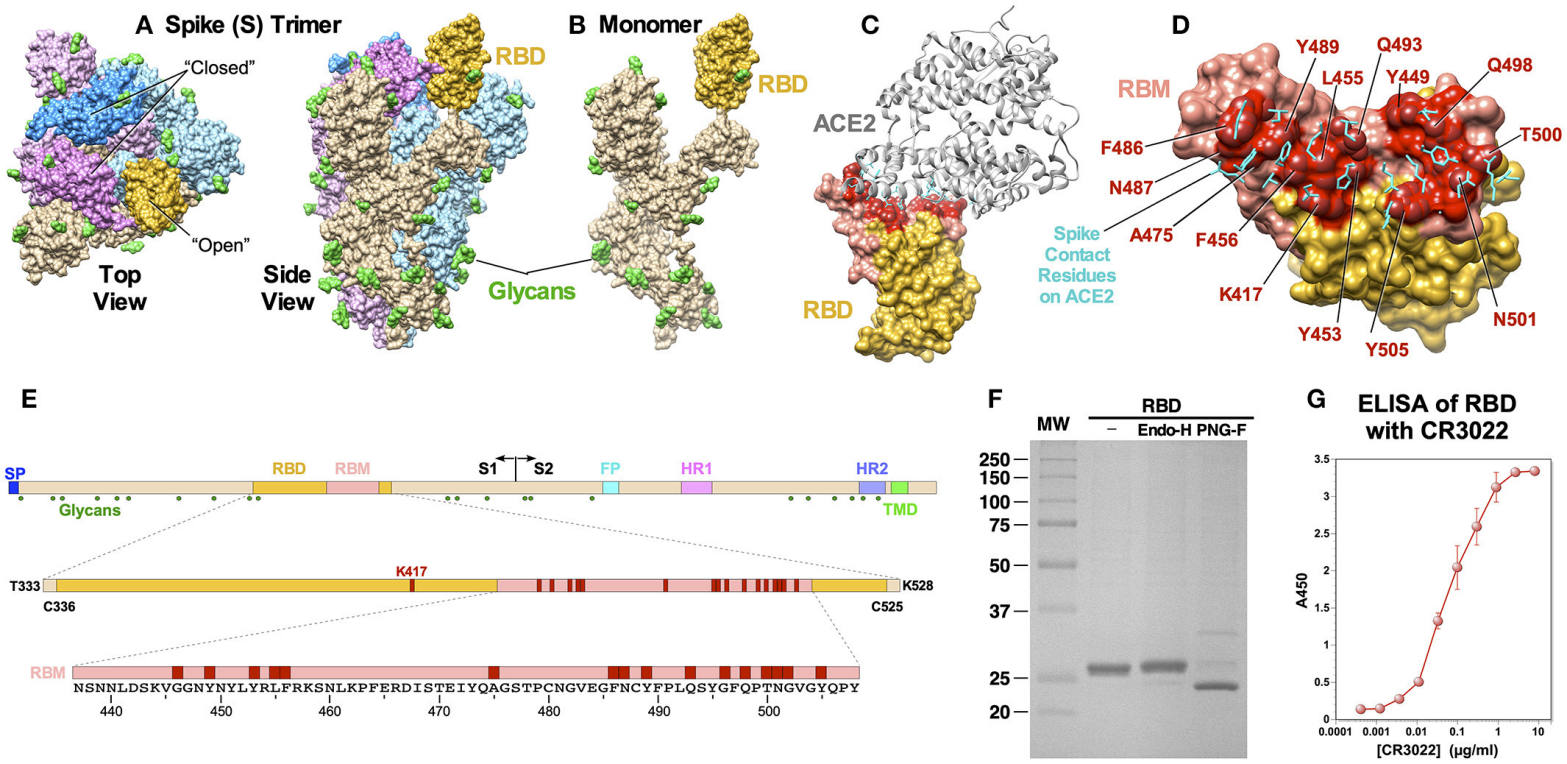
Structural features of SARS-CoV-2 S protein and RBD immunogen. **(A)** A cryo-EM structure of a trimer (PDB: 6VSB). Top and side views are shown, and the “open” and “closed” conformational states of the RBD are indicated. Three monomers are shown in different colors with the RBD portions highlighted in darker shades. Glycans (N-acetylglucosamine) are shown in green. **(B)** A monomeric S protein in an “open” state. **(C)** A cocrystal structure of the RBD with ACE2 (PDB: 6M0J). The RBM is shown in pink and the ACE2 binding residues are highlighted in red. **(D)** Details of amino acid residues involved in ACE2 binding. Side chains on ACE2 that bind the RBD are shown in cyan. **(E)** Schematic diagrams of S protein, RBD and RBM showing major functional domains and features. The RBM is shown in pink and ACE2-contact residues are shown in red. Glycosylation sites are shown as green hexagon. **(F)** SDS-PAGE of purified RBD and after treatment with endoglycosidase H and PNGase F. **(G)** ELISA showing binding of the RBD to neutralizing mAb CR3022.

Cryo-EM structures of SARS-CoV-2 S protein as well as cocrystal structures of the receptor binding domain (RBD) of the protein bound to ACE2 have been solved (5–8). S protein functions as a homotrimer (Figure 1A) and it is highly glycosylated. Glycosylation of all 22 potential N-linked glycosylation sites have been confirmed for SARS-CoV-2 (9). The RBD folds independently into a globular structure away from the rest of the S protein (Figure 1B). It exists in two different conformations on the trimer: “open” and “closed” states (Figure 1A). While it is in the “open” state, it can bind ACE2. Cocrystal structures of the RBD and ACE2 clearly identified amino acid residues on both proteins critical for binding (Figures 1C–E). Within the RBD, binding of ACE2 is mediated mostly by amino acid residues within a short linear segment called the receptor binding motif (RBM) (Figures 1D,E).

Many studies have shown that subunit protein antigens based on the RBD can elicit neutralizing antibodies (nAbs) against SARS-CoV (10–13). In this study, we examined immunogenicity of a SARS-CoV-2 recombinant RBD subunit protein vaccine candidate in mice using three different adjuvants. We focused on the humoral immune responses since nAbs are considered to be the major immune correlates of protection (14). In particular, we monitored on the potency, induction kinetics and durability of nAbs. The results indicate that potent, long-lasting nAbs can be induced using the RBD, that adjuvants can profoundly affect antibody responses, and that RBD-based subunit protein is a highly promising vaccine candidate against COVID-19.

## Results

### Immunogen Design, Production, and Characterization

The RBD contains a single N-linked glycosylation site at N^343^. To generate an RBD immunogen that is as native as possible, we constructed a plasmid encoding the RBD to be expressed in mammalian cells. The immunogen encompasses amino acids from T^333^ to K^528^, which are located three residues upstream or downstream of two cysteine residues, C^336^ and C^525^, respectively, that form a disulfide bridge and structurally define the RBD. The RBD gene is preceded by a signal peptide for secretion and followed by a hexahistidine tag (6×H) for purification. The recombinant RBD protein was transiently expressed in HEK 293F cells and purified easily from cell culture supernatant through a single-step Ni-NTA affinity chromatography (Figure 1F). The protein migrated slower than the expected molecular weight of ~24 kD, indicating possible glycosylation. Indeed, treating the protein with PNGase F increased mobility. The protein was largely resistant to endoglycosidase H, suggesting that protein is likely glycosylated with mostly complex sugars, rather than high mannose. Correct folding of the RBD was confirmed by demonstrating its reactivity to a mAb CR3022 (15, 16) (Figure 1G). A cocrystal structure of CR3022 bound to SARS-CoV-2 RBD (17) and its neutralizing activity against the virus (18) have been reported.

### Antibody Responses Against the RBD in Mice

**Figure 2:**
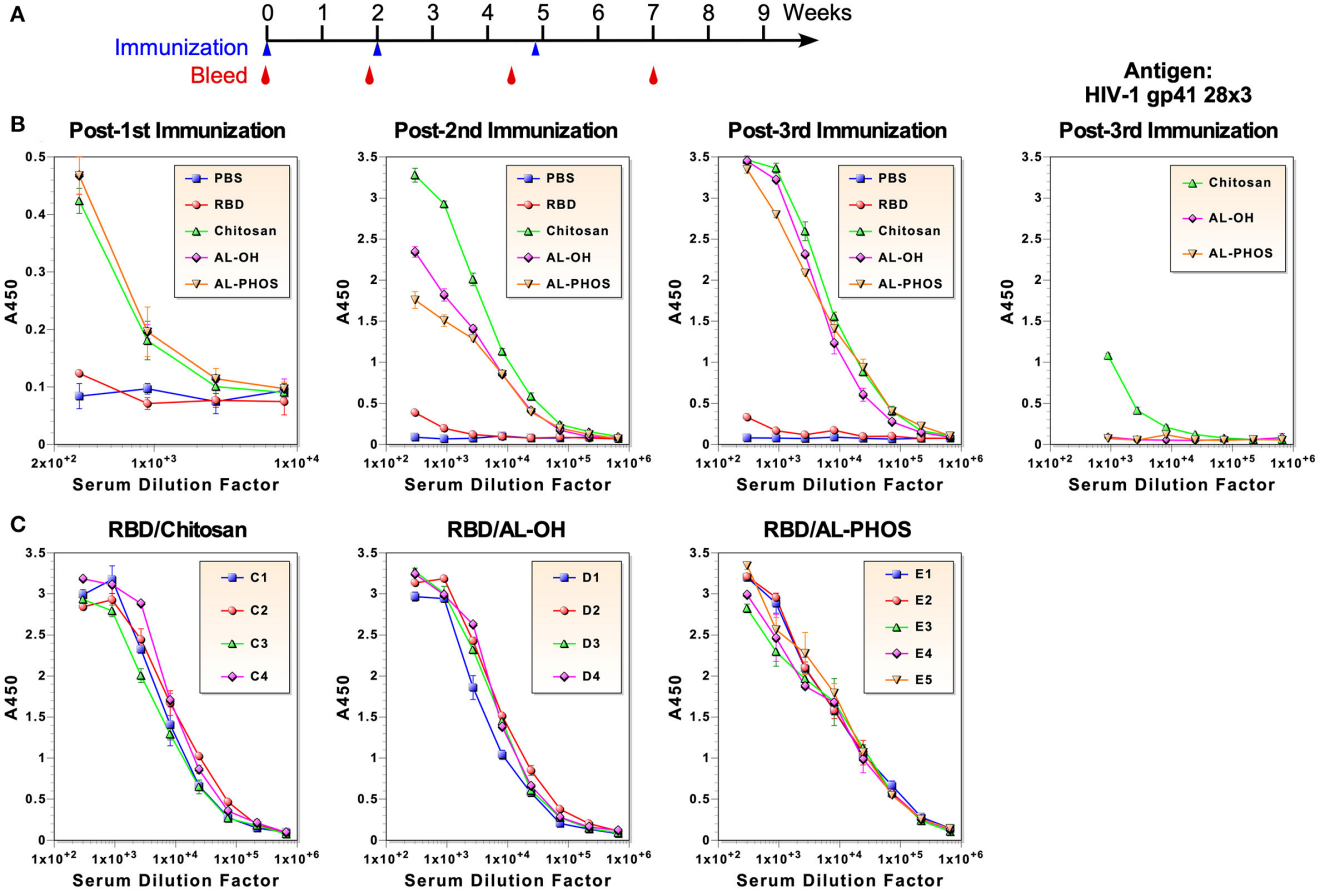
Antibody responses against the RBD immunogen. **(A)** Immunization and blood collection schedule. **(B)** ELISA of pooled antisera collected about 2 weeks after each immunization. ELISA was also done using HIV-1 gp41-28×3 to assess antibodies elicited against the 6×His tag (right panel). **(C)** ELISA of antisera from individual animals after the third immunization.

To evaluate immunogenicity of the RBD, 5–6 weeks old mice were immunized intraperitoneally three times on weeks 0, 2, and ~5. Animals were bled before and about 2 weeks after each immunization (Figure 2A). Five groups of mice (4 or 5 mice per group) were immunized: (1) PBS (mock immunization control), (2) RBD only without an adjuvant, (3) RBD formulated with Alhydrogel (aluminum hydroxide; ALH), (4) RBD with Adju-Phos (aluminum phosphate; ADP), and (5) RBD with Zn-chitosan (CHT). Chitosan is a biodegradable, cationic polysaccharide derived from chitin by partial deacetylation. We selected aluminum-based adjuvants because they induce Th2 responses, which are important for eliciting strong humoral immunity, and because they are FDA-approved for human use. Although Zn-chitosan is not FDA-approved, we included it for comparison because we have used it successfully to induce potent antibody responses (19–21).

To examine anti-RBD antibody responses, ELISA was done using serum samples after each immunization (Figure 2B). Due to limited amount of blood that could be collected from each mouse, pooled sera from each group were used for all assays, unless noted otherwise. After the first immunization with 30 μg, all three groups of mice immunized with adjuvants mounted similar antibody responses with end-point titers of ~3,000. No antigen-specific antibodies were detected in mice immunized without an adjuvant. As expected, robust anamnestic responses were observed after the second immunization with 20 μg (end-point titers of ~2 × 10^5^). The antibody response was particularly stronger in mice immunized with RBD adjuvanted with Zn-chitosan. Antibody response was barely detected in mice immunized without any adjuvant. After the third immunization (20 μg), all vaccine groups immunized with adjuvant elicited similar antibody titer of ~6 × 10^5^. Even after three immunizations, antibody response was barely detectable in the RBD group without an adjuvant. To assess possible animal-to-animal variations in antibody responses, ELISA was also done using serum samples from individual animals collected about 2 weeks after the third immunization (Figure 2C). Results showed that there were no major differences in antibody levels between animals within each group.

Since ELISA was done using the RBD immunogen, there was a possibility that some of the antibody responses could be against the C-terminal 6×His tag. To assess the level of potential antibodies against the tag, we did ELISA against a heterologous protein that also has a C-terminal 6×His tag (HIV-1 gp41-28×3), which we previously described (22). As shown in Figure 2B (far right panel), a low level of antibodies was detected in the Zn-chitosan group (<3% of the antibody responses against the RBD immunogen). Interestingly, Alhydrogel and Adju-Phos groups did not mount any detectable antibodies against the 6×His tag.

### Assessment of Virus Neutralizing Activity

**Figure 3:**
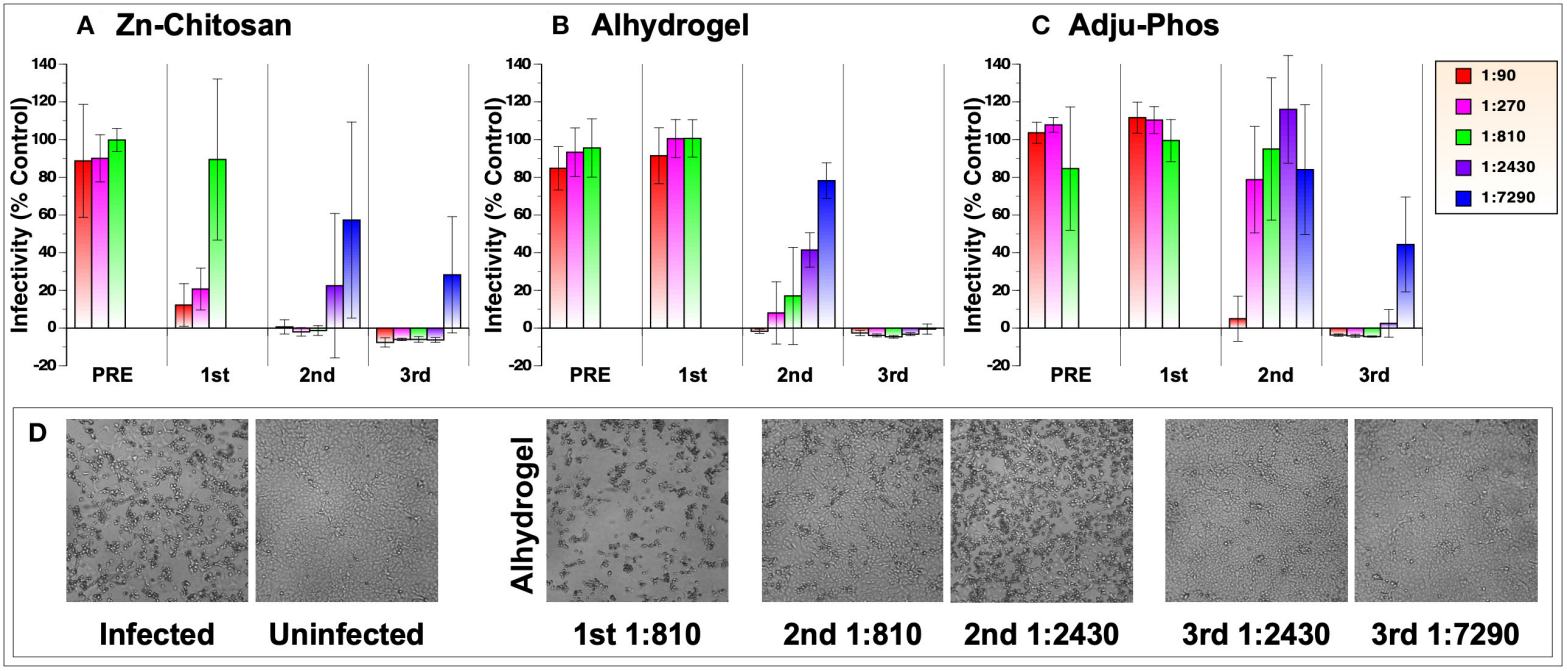
SARS-CoV-2 neutralization assay. Vero E6 cells (2 × 10^4^ cells/well) in 96-well-plates were infected with 50 pfu with various dilutions of pooled sera collected before (PRE) or after three immunizations (indicated as 1st, 2^nd^, and 3rd). Virus infection was monitored by CyQuant LDH (lactate dehydrogenase) Cytotoxicity Assay, which measures the level of cytosolic LDH released into the culture medium when cells die from virus infection. The assay was done in triplicate. Results from three vaccine groups are shown: **(A)** Zn-chitosan, **(B)** Alhydrogel, and **(C)** Adju-Phos. **(D)** Brightfield photomicrographs of cell cultures from the neutralization assay. Results from the Alhydrogel group (for indicated immunizations and dilutions) are shown as examples.

Virus neutralizing activity of antibodies was measured against 50 plaque forming units (50 PFU) of live, infectious SARS-CoV-2 in Vero E6 cells. This was done by monitoring cellular cytotoxicity microscopically and by quantifying release of lactate dehydrogenase (LDH) into cell culture medium. Comparison of neutralizing activity of three vaccine groups with different adjuvants is shown in Figure 3A. Overall, Zn-chitosan group exhibited the best neutralizing activity, followed by Alhydrogel group and then Adju-Phos groups. After the first immunization, 80% neutralization titer (NT_80_) was ~270 for the Zn-chitosan group (Figure 3A), which is higher than average neutralization titers observed in convalescent sera (~160) (23, 24). No neutralizing activity was observed in either Alhydrogel or Adju-Phos group at the dilutions tested (Figures 3B,C, respectively). After the second immunization, neutralizing activity increased nearly 10-fold to NT_80_ of ~2,430 for the Zn-chitosan group. NT_80_ in Alhydrogel group was ~810 while NT_80_ was between 90 and 270 for Adju-Phos group. The neutralizing activity correlated well with RBD-binding antibody titers (Figure 2B). After the third immunization, all three groups mounted strong nAb responses, especially the Alhydrogel group with NT_100_ of at least 7,290. It should be noted, however, that both RBD-binding antibodies (Figure 2B) and nAbs (Figure 3A) did not increase substantially after the third immunization for the Zn-chitosan group. We suspect this is most likely due to the presence of high level of preexisting antibodies at the time of third immunization in this group. Of note, we frequently observed higher standard deviations for neutralization assays near end point dilutions of serum samples. We suspect this variation at high serum dilutions is due to near, but not always total neutralization of the virus at these low serum concentrations where nAbs are limited. In instances with complete neutralization at these dilutions, no infectivity is observed. However, if even a small amount of virus remains unneutralized (e.g., 5%), it can propagate, yielding high rates of infectivity. This “all-or-none” phenomenon is more pronounced at high serum dilution, and likely underlies the high variability seen at these dilutions.

Microscopic observations of the cytopathic effects paralleled the neutralizing activity assessed by the LDH assay (Figure 3D). As examples, photomicrographic images of infected or uninfected cells in the absence or presence of various dilutions of sera after the first, second or third immunizations from the Alhydrogel group are shown.

**Figure 4:**
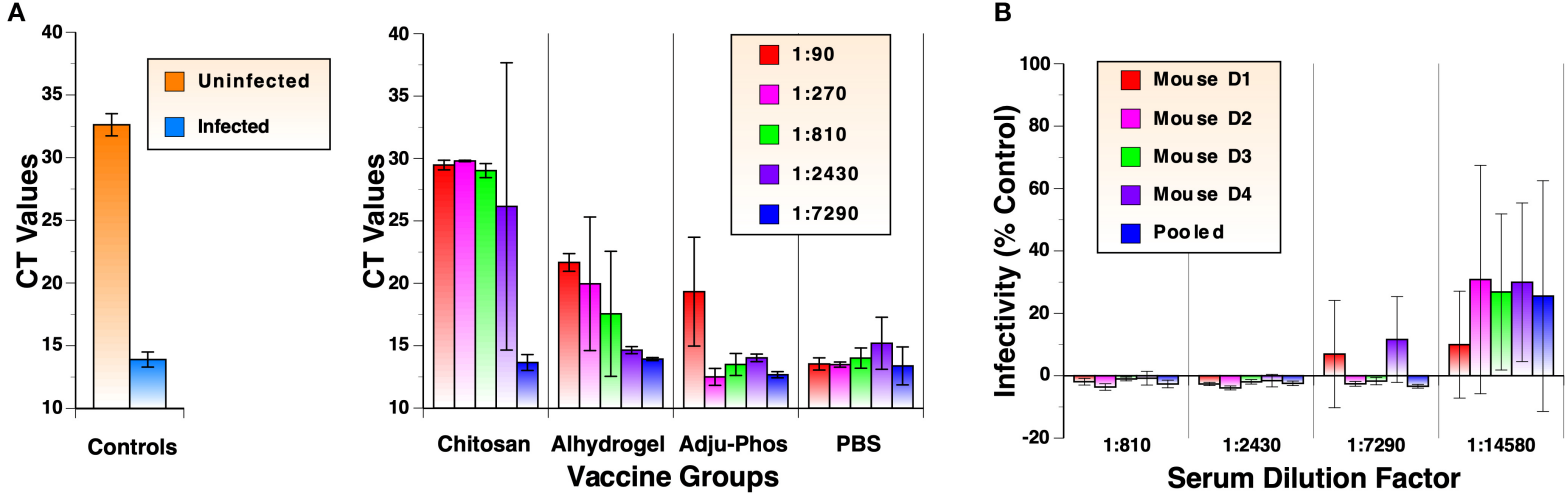
Detailed analyses of neutralizing antibody responses. **(A)** RT-qPCR analyses of virus neutralization assay. Cell culture supernatants from neutralization assays using antisera after the 2nd immunization were evaluated in triplicate. Viral RNA was harvested from culture media of virus-infected cells. Following reverse transcription, qPCR was carried out using IDT 2019-nCoV RUO Kit. **(B)** Extended nAb titration of antiserum from individual mouse in the Alhydrogel group after 3rd immunizations. Pooled sera were also analyzed for comparison.

To validate neutralization assay results using the LDH cytotoxic assay, viral RNA in cell culture media from the assay were analyzed in parallel by quantitative RT-PCR. Results of the analyses using samples after the 2nd immunization are shown in Figure 4A. Cycle threshold (CT) values for samples from infected and uninfected cells were 13.9 and 32.6, respectively. CT values for samples from mock-immunized PBS group ranged between 13.4 and 15.2, indicating no inhibition of virus replication. Results from the RT-PCR assay correlated very well with those of LDH cytotoxic assay. The rank order of neutralizing activity was Zn-chitosan>Alhydrogel>Adju-Phos. Complete neutralization of viruses at 1:90, 1:270, and 1:810 dilutions for Zn-chitosan group was confirmed by RT-PCR analysis. Slightly lower CT values for these samples (29.5, 29.8, and 29.0, respectively) compared to uninfected sample (32.6) likely represent presence of non-infectious viruses from the inoculum.

As shown in Figure 3B, mice in the Alhydrogel group induced NT_100_ of at least 7,290. Although this was achieved after three immunizations, this is highly significant because the adjuvant is already FDA-approved for human use. To quantify nAb levels more precisely, neutralization assays were repeated for this group with more diluted antisera from individual mice. As shown in Figure 4B, all mice mounted NT_70_ of ~14,580.

### Durability of Antibody Responses

**Figure 5:**
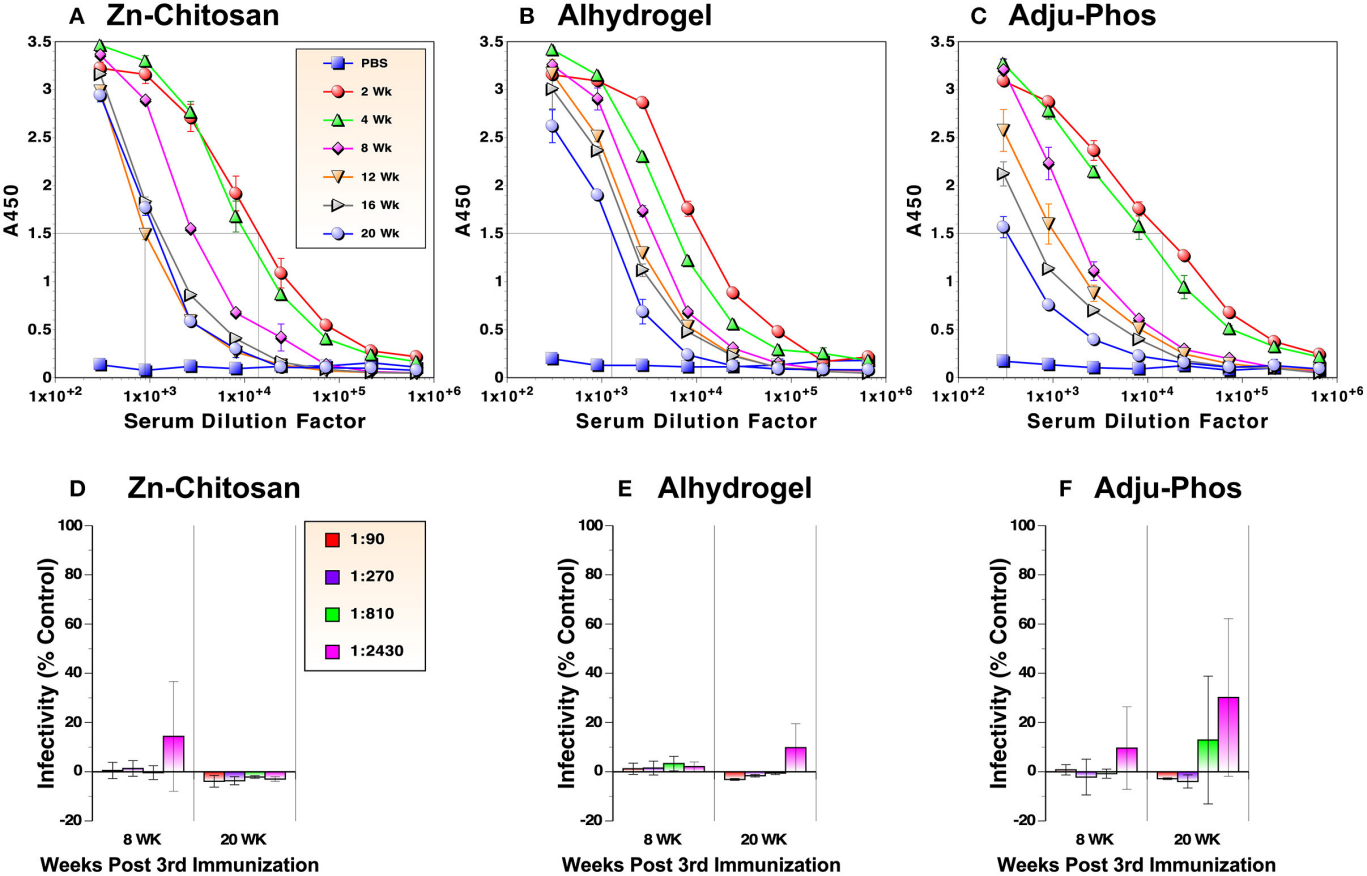
Durability of antibody responses against the RBD. RBD-binding antibody levels were monitored by ELISA in duplicate **(A–C)**. Antiviral activity was assessed by neutralization assays against live, infectious SARS-CoV-2 in triplicate **(D–F)**. Time indicates weeks after the third immunization.

One of the important criteria for assessing vaccine efficacy is the longevity of protection after vaccination. As such, we examined durability of antibody levels against the RBD. Serum samples were collected from mice every 2 or 4 weeks after the third immunization for 5 months and evaluated by ELISA. For the Zn-chitosan group, antibody levels decreased during the first 3 months (~14-fold from 2 to 12 weeks) but stabilized afterwards (Figure 5A). For the Alhydrogel group, the antibody level decreased more gradually during the 5-month period (~10 fold from 2 to 20 weeks) (Figure 5B). For the Adju-Phos group, the decline was more drastic (~43 fold; Figure 5C).

We also assessed nAb levels at 8 and 20 weeks after the third immunization. For both Zn-chitosan and Alhydrogel groups, near complete neutralization was observed at 1:2,430 dilution even after 20 weeks (Figures 5D,E, respectively). Even for the Adju-Phos group, NT_80_ was > 810 (Figure 5F).

### Evaluation of Antibody Avidity

**Figure 6:**
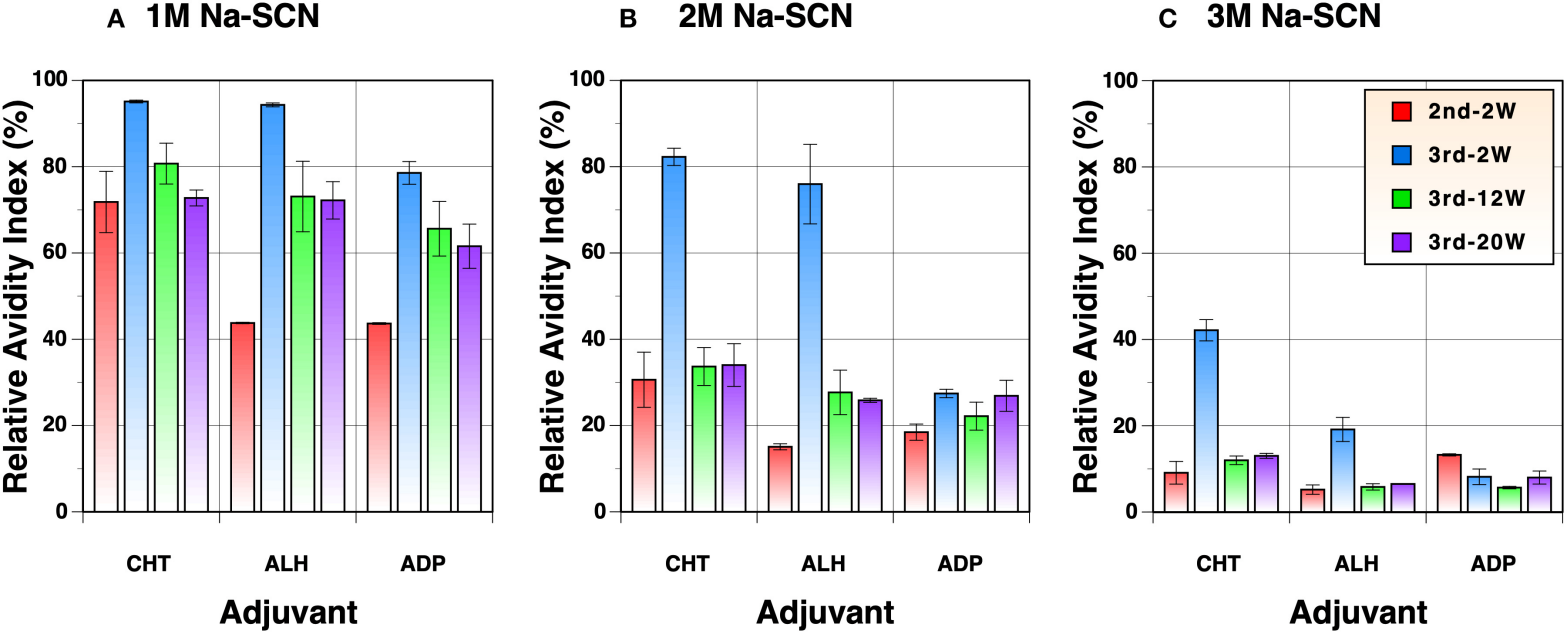
Avidity of antibodies against the RBD. ELISA was done in the presence of **(A)** 1M, **(B)** 2M or **(C)** 3M NaSCN and compared to without to determine relative avidity index. Serum samples collected 2 weeks after the second and 2, 12, or 20 weeks after the third immunizations were evaluated at 1:1,800 dilution.

Although the antibody titers were virtually identical among the three groups vaccinated with different adjuvants after the first immunization (Figure 2B), the only group that exhibited neutralizing activity at dilutions tested was the Zn-chitosan group (Figure 3A). The differences in the neutralizing activity were more pronounced after the second immunization, especially when compared to the Adju-Phos group (Figure 3). This difference could be due in part to differences in the epitopes being targeted as well as affinity/avidity of antibodies. As such, we assessed avidity of the antibodies at various times after immunization by doing ELISA in the absence or the presence of different concentrations of sodium thiocyanate (NaSCN). Overall, antibodies induced in Zn-chitosan group exhibited higher avidity, followed by Alhydrogel and Adju-Phos (Figure 6). The observed avidity generally correlated with neutralizing activity. The avidity increased substantially after the third immunization compared with that after the second immunization, especially for the Zn-chitosan and Alhydrogel groups. The high avidity was striking especially for the Zn-chitosan group with >40% of the antibodies able to bind the RBD even in 3 molar NaSCN.

### Examination of Immunogenic Linear Epitopes

**Figure 7:**
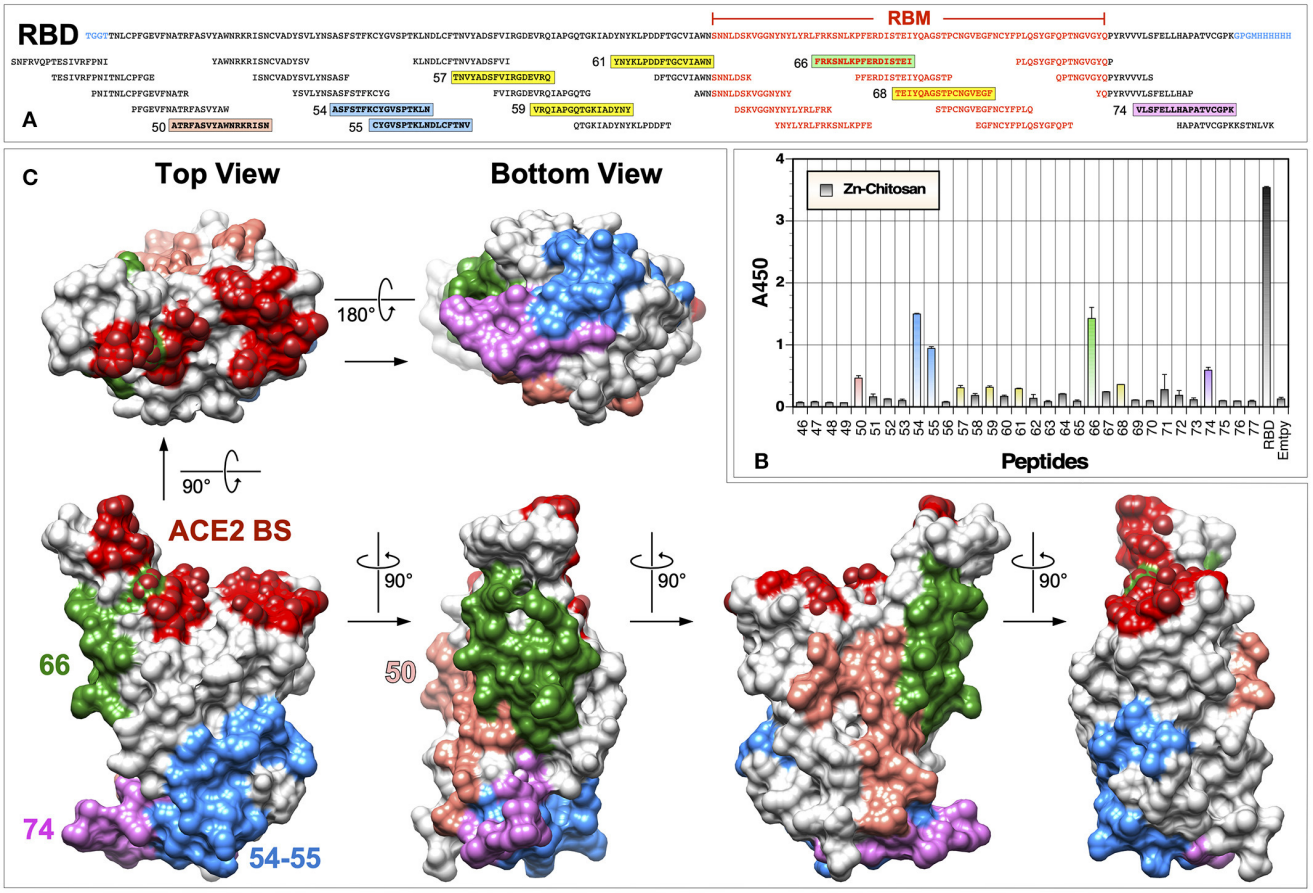
Identification of immunogenic epitopes using overlapping peptides. **(A)** Sequence of the RBD and overlapping peptides (obtained from BEI Resources) used in ELISA. Amino acid residues located within the RBM are indicated in red text. Immunogenic epitopes are highlighted in colored boxes. **(B)** ELISA was done using serum samples (1:300 dilution) from mice immunized with Zn-chitosan, collected 6 weeks after the third immunization. Two hundred nanograms of peptides were coated in each well. RBD was used as a positive control. A negative control without any peptides is indicated as “Empty.” No immunoreactive peptides were identified using serum samples from Alhydrogel or Adju-Phos groups. Peptide numbers represent those from the 181-peptide array of the S protein. **(C)** Locations of the five most immunogenic linear epitopes are mapped onto the RBD structure (PDB: 6M0J).

Although vaccines must elicit high antibody titers, what is really important for their protective efficacy is whether elicited antibodies can target critical neutralizing epitopes and inhibit infection either by blocking binding to cellular receptors or by preventing conformational changes that are required for the virus entry process (e.g., membrane fusion). To begin to assess immunogenic epitopes on the RBD, we conducted ELISA with a panel of 17-mer overlapping peptides (10 a.a. overlap) that cover the entire length of the RBD immunogen (Figure 7A). From this analysis, several linear epitopes were identified for the Zn-chitosan group (Figure 7B). The three most immunogenic epitopes were ^372^ASFSTFKCYGVSPTKLN^388^ (#54), ^379^CYGVSPTKLND-LCFTNV^395^ (#55) and ^456^FRKSNLKPFERDISTEI^472^ (#66). Two other epitopes with lower reactivity were ^344^ATRFASVYAWNRKRISN^360^ (#50) and ^512^VLSFELLHAPATVCGPK^528^ (#74). The locations of these five peptides on the RBD are shown in Figure 7C. Of these peptides, only peptides #66 was located within the RBM. There were a few other weakly reactive peptides, including ^393^TNVYADSFVIRGDEVRQ^409^ (#57), ^407^VRQIAPGQTGKIADYNY^423^ (#59), ^421^YNYKLPDDFTGCVIAWN^437^ (#61) and ^470^TEIYQAGSTPCNGVEGF^486^ (#68) (Figure 7A, highlighted in yellow).

One major unexpected result was that we were unable to detect any immunoreactive peptides when we used sera from animals immunized with Alhydrogel or Adju-Phos. Although this is likely due to genuine differences between immune responses elicited using different adjuvants, it could also be due to inefficiencies of the ELISA using 17-mer overlapping peptides. First, the peptides could be too short to fold into structures that would resemble native structures on the RBD. Second, peptides could be adhered to ELISA plates in a manner that might not allow binding of antibodies elicited against them on the RBD. Even for detecting immunoreactive peptides for the Zn-chitosan group, we had to use 200 ng of peptides in each well and 1:300 dilution of sera.

**Figure 8:**
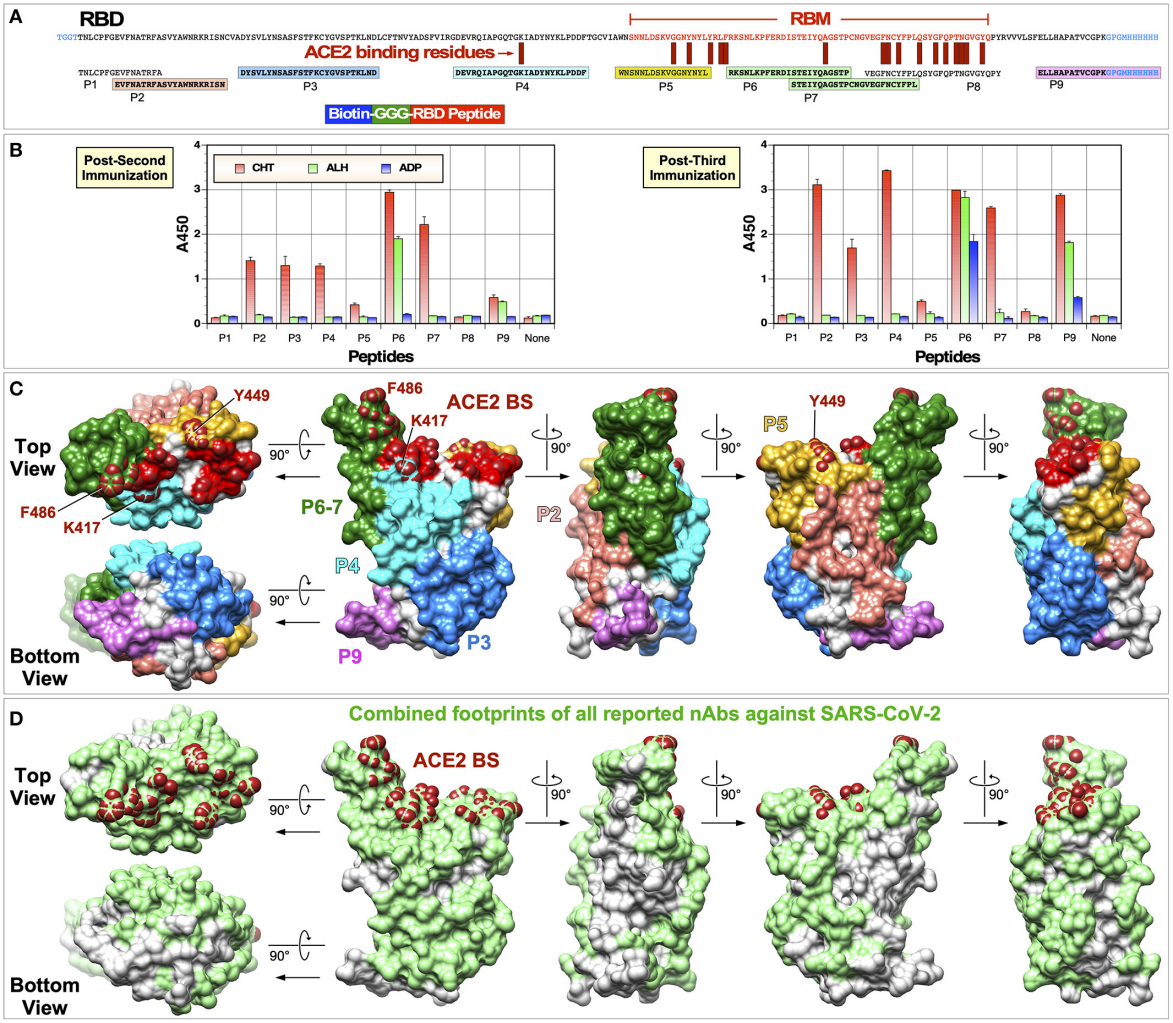
Identification of immunogenic epitopes using biotinylated peptides. **(A)** Sequences of the RBD and biotinylated peptides used are shown. Amino acid residues located within the RBM are indicated in red text. Immunogenic epitopes are highlighted in colored boxes. **(B)** ELISA was done using serum samples collected 2 weeks after the second or third immunization (1:300 dilution). ELISA plates were coated with streptavidin (300 ng per well) and 100 ng peptides were attached to streptavidin. Negative controls without any peptides are indicated as “None.” **(C)** Locations of immunogenic peptides are mapped onto the RBD structure (PDB: 6M0J). **(D)** Combined footprints of 32 neutralizing mAbs with known crystal structures are shown.

To improve efficiency of detecting immunoreactive peptides, we used nine peptides shown in Figure 8A. These peptides are different from the overlapping peptides shown in Figure 7A in following ways. First, the peptides were designed based on their structure and surface exposure on the RBD. As such, they are of different lengths and do not cover the entire sequence of the RBD. Second, these peptides are preceded by three glycine residues and they are biotinylated at the N-terminal amine group. This allows the peptides to bind to streptavidin-coated ELISA plates. The three glycine residues serve as a spacer between streptavidin and the RBD portion of peptide. Thus, the RBD peptides should be more readily accessible to bind antibodies. As such, we hypothesized these peptides should be better suited for identifying immunogenic epitopes.

ELISA was done using the new peptides with serum samples collected 2 weeks after the second or third immunization (Figure 8B). As hypothesized, we were able to detect immunoreactive peptides more readily. Overall, results were in good agreement with what was observed with overlapping 17-mer peptides. First, serum samples collected from the Zn-chitosan group reacted to more peptides than sera from the other two groups. While sera from Zn-chitosan group reacted against peptides P2, P3, P4, P5, P7, and P9 after three immunizations, sera from Alhydrogel and Adju-Phos groups reacted only against P6 and P9 peptides. Second, both assays identified similar immunogenic peptides: P2 (#50), P3 (#54 and #55), P4 (#59), P5 (#59), P6 (#66), P7 (#68), and P9 (#74). It should be noted that peptide ELISA shows relative immunogenicity of peptides to each other, not to the RBD since the molar amounts of peptides we used were ~6×fold higher than the amount of RBD, and the serum concentration was >3×fold higher than that used to observe similar absorbance values for the RBD.

For the Zn-chitosan group, the two most immunogenic linear epitopes were within P6 (^457^RKSNLKPFERDISTEIYQAGSTP^479^) and P7 (^469^STEIYQAGSTPCNGVEGFNCYFPL^492^). They are situated in the middle of the RBM and contain residues that are critical for binding ACE2 (Figure 8A). However, these peptides mostly face away from the ACE2 binding site (Figure 8C). Depending on which face of the peptide structure antibodies bind, they may or may not exhibit neutralizing activity. More detailed epitope mapping analyses and characterization of antibodies at the monoclonal level would be needed.

Most of the linear immunogenic peptides were distant from the actual ACE2 binding site (Figure 8C). However, these peptides, with the exception of peptide P9, form parts of the RBD surface that overlap footprints of known nAbs against SARS-CoV-2 (Figure 8D). For example, peptide P3 is well away from the ACE2 binding site. However, several neutralizing monoclonal antibodies (mAbs) bind this region, including S2A4 (25), H014 (26), CR3022 (18), EY6A (27)m and H11-H4 (28). Another example is mAb S309 (29), footprint of which lies on peptide P2. Thus, antibodies that bind to these peptides could exhibit neutralizing activity.

Peptide P8, which is situated at the C-terminal end of the RBM and contains the greatest number of residues that make direct contact with ACE2, was not reactive to any of the antisera. This does not necessarily mean that nAbs that bind the ACE2 binding site were not elicited. It is highly likely that the P8 peptide is unable to fold into a conformation that resembles the native structure found on the intact RBD. Peptide P5, which is situated at the N-terminal end of the RBM and contains three residues that contact ACE, was weakly reactive.

## Discussion

To develop a vaccine, two major factors have to be considered, an immunogen and a vaccine modality. They are decided largely based on what immune correlates of protection are, how best the protective immunity can be induced, and how easily or cost-effectively vaccine candidates can be produced. For viruses, neutralizing antibodies are critical correlates of protection. No doubt, this is the reason why all COVID-19 subunit vaccine candidates being evaluated are based on the spike glycoprotein. It is also the reason why induction of potent and durable neutralizing antibody responses will be critical for the protective efficacy of COVID-19 vaccines.

During the past 11 months, significant global efforts have been made toward developing COVID-19 vaccines using different vaccine platforms. They include vaccines based on inactivated viruses, nucleic acids (DNA and RNA), viral vectors, and recombinant subunit proteins. Several vaccine candidates are already in Phase 3 clinical trials, including those by Moderna/NIH (mRNA), BioNTech/Pfizer (mRNA), CanSino Biologics (Adenovirus 5), University of Oxford/AstraZeneca (ChAdOx1), Janssen/Johnson and Johnson (Adenovirus 26), Wuhan Institute of Biological Products/Sinopharm (inactivated virus), Beijing Institute of Biological Products/Sinopharm (inactivated virus), and Sinovac (inactivated virus).

Our efforts to develop a subunit protein vaccine based on the RBD stems largely from prior vaccine development efforts made against SARS-CoV. Since the emergence of SARS-CoV in 2002, various strategies have been explored. They include inactivated viruses (30–35), a live-attenuated virus (36), DNA (37–40), and a viral vector vaccine (41). Compared with these vaccine modalities, subunit protein vaccines based on the RBD have shown to induce higher nAb titers. Different RBD-based immunogens, either alone (12) or fused to IgG Fc (13), have been produced in various recombinant protein expression systems, including mammalian cell lines (293T, CHO), insect cells (Sf9), yeast and *E. coli* (10, 12, 42). Their immunogenic properties have been evaluated in different animal models using different adjuvants and immunization routes. To summarize, it has been demonstrated that (i) RBD can sufficiently induce nAbs; (ii) RBD expressed in 293T cells elicited higher nAbs than RBD expressed in Sf9 or *E. coli* (12); and (iii) RBD-based vaccines can induce long-term neutralizing activity and protective immunity (11). Importantly, it has been shown that potent and persistent antibody responses against the RBD exist in recovered patients (42). Potential advantages of RBD-based vaccines over the use of the entire S protein have been discussed (43).

In this study, we evaluated immunogenicity of a glycosylated, monomeric RBD-based subunit protein immunogen (T^333^ to K^528^) produced in 293F human cells. Three different adjuvants were compared. Using Alhydrogel, an FDA-approved adjuvant for clinical use, we did not detect nAbs after the first immunization, at least at 1:90 dilution. However, two immunizations induced NT_80_ of ~810 (NT_50_ of >2,430) against live infectious SARS-CoV-2, which is significantly greater than titers observed in convalescent sera in recovered patients (23, 24). One additional immunization induced remarkably high NT_70_ of ~14,580. The neutralizing antibody response was highly durable. Despite gradual decline, NT_80_ remained >2,430. The antibody responses were substantially weaker using Adju-Phos with respect to all major parameters monitored (i.e., kinetics of nAb induction, titer of nAbs, durability, and avidity).

In contrast to alum-based adjuvants, Zn-chitosan was able to induce potent nAbs even after a single immunization with NT_80_ of ~270 despite similar antigen-binding antibody levels. The NT_80_ increased almost 10-fold to ~2,430 after the second immunization, about 3-fold higher than the Alhydrogel group. However, after the third immunization, the neutralizing activity increased only about 3-fold, compared to >81-fold for the Alhydrogel group. We suspect this is most likely due to interference by high levels of pre-existing antibodies after the second immunization, similar to maternal antibody interference of vaccine efficacy. In this regard, a longer vaccination interval to allow antibody waning could have improved boosting efficiency and resulted in greater neutralizing activity. In this study, we used Zn-chitosan as a yardstick to gauge immunogenic potential of our RBD immunogen. The results indicate that RBD is inherently immunogenic and that it may be possible to elicit stronger antibody responses than what we observed using Alhydrogel if we were to use more potent adjuvants that are already approved by the FDA.

To date, results from many preclinical and clinical vaccine studies have been reported. Protective efficacy of two β-propiolactone-inactivated virus vaccines [BBIBP-CorV (44) and PiCoVacc (45)] have been evaluated in non-human primates. Macaques were immunized two or three times with BBIBP-CorV or PiCoVacc, respectively. Although both vaccines were able to protect animals, nAb titers were relatively weak (NT_50_ of 256 and 50, respectively). One important consideration is that animals were challenged <10 days after the final immunization. Thus, the long-term protective efficacy of these vaccine candidates is currently unknown.

A preliminary report from the Phase 1 clinical trial by Moderna indicated that an RNA vaccine that encodes a stabilized prefusion trimeric spike protein can elicit both cellular and humoral immune responses (23). The vaccine required two doses to elicit binding and neutralizing antibody titers that were comparable to those observed in human convalescent sera. NT_80_ of 340 and 654 were elicited using 25 and 100 μg doses, respectively. The same vaccine in 100 μg dose was able to induce high levels of nAbs (NT_50_ of 3,481) and significantly reduce viral load in rhesus macaques against virus challenge 4 weeks post last immunization (46). Similarly, DNA vaccines encoding various segments of S protein also reduced viral load by over 3 logs in bronchoalveolar lavage and nasal mucosa (47). However, neutralizing antibody titers against infectious SARS-CoV-2 were relatively low (median titer of 74).

Vaccines based on viral vectors are also making significant progress. Macaques immunized with chimpanzee adenovirus vector encoding S protein (ChAdOx1mCoV-19) also exhibited similar protection with neutralizing antibody titers ranging 10–160 (48). The same vaccine induced median NT_80_ of 136 after two immunizations in a phase 1/2 trial (49). Similarly, Janssen/Johnson and Johnson's adenovirus 26-based vector that expresses stabilized trimeric S protein induced only NT_50_ of 113 in macaques but was protective against 1 × 10^5^ TCID_50_ SARS-CoV-2 challenge (50). Preliminary data from their Phase 1/2a study are currently being peer reviewed.

Protective efficacy of a recombinant RBD produced in Sf9 insect cells using a recombinant baculovirus has also been evaluated in macaques (51). Animals were immunized twice intramuscularly 7 days apart with 20 or 40 μg of recombinant RBD. Although relatively low levels of nAbs were induced (NT_50_ of ~100), the animals were protected from SARS-CoV-2 challenge 3 weeks after the last immunization.

There have been a number of efforts to enhance immunogenicity of the RBD. One strategy was to fuse the protein to the Fc domain of human IgG1 (RBD-Fc) (52). Mice were immunized subcutaneously twice, 14 days apart, with 10 μg of antigens using MF59 as an adjuvant. Mice immunized with RBD-Fc mounted much higher nAbs against live SARS-CoV-2 with NT_100_ of 25 compared to only 5 for RBD. Another strategy that has been evaluated to increase immunogenicity of the RBD is to generate a single chain dimeric RBD by cloning two RBD in tandem (53). Mice were immunized with 10 μg of monomeric or dimeric RBD twice using AddaVax as an adjuvant. Compared to monomeric RBD which only induced NT_50_ of only 128 or 256 in two of eight animals, dimeric RBD induced average NT_50_ of 3,008. Another strategy that has been evaluated is to deliver RBD as a particulate form using liposomes (54). Here, a recombinant RBD with a histidine tag was incorporated onto liposomes containing cobalt-porphyrin-phospholipid (CoPoP). Mice were immunized twice intramuscularly with 100 ng of RBD using monophosphoryl lipid A (MPLA) or MPLA/QS21 as adjuvants. NT_50_ of 1,280 were induced in both groups, compared to NT_50_ < 160 for the same immunogen adjuvanted with Alum. They were able to induce similar level of nAbs in rabbits using 20 μg of the RBD, but only when QS21 was used, compared to NT_50_ of ~320 using Alum.

Not surprisingly, RBD immunogen can be delivered by mRNA platform as well (55). Mice immunized twice, 4 weeks apart, with 30 μg of RNA elicited NT_50_ of 540 against infectious SARS-CoV-2. Importantly, immunization with RBD induced significantly higher nAb titer than a comparable RNA vaccine encoding S1 domain of the spike protein. Most recently, results of Phase 1/2 evaluation of BioNTech/Pfizer's RNA vaccine (BNT162b1) that encodes trimeric RBD was published (56). The vaccine was administered intramuscularly twice, separated by 21 days. NT_50_ of about 437 was induced against infectious SARS-CoV-2 using 30 μg dose.

Compared with most other COVID-19 vaccine studies, especially those that evaluated immunogenicity of the RBD, we were able to induce higher titers of nAbs. While this could be due to inherent differences between immunogens (e.g., RBD vs trimeric S protein) and/or vaccine delivery platforms (e.g., subunit protein vs. RNA or viral vector), there could be other possible reasons (e.g., antigenic dosage, immunization routes, adjuvants, vaccination schedule, and animal model used). Since no two vaccine regimens are exactly identical, it is not simple to determine which immunogen or vaccine delivery platform is better. In addition, it should be noted that there are subtle differences between virus neutralization assays (e.g., assay methodology and amounts of infectious units used). Standardizing the methodology or having a common positive control could facilitate comparing different vaccine candidates.

Most vaccine studies that have been conducted thus far used two-immunization regimens. While two immunizations might be sufficient to elicit nAb titers that are greater than the levels observed in convalescent sera, the results from our study clearly demonstrate that a third immunization can substantially increase antibody avidity (Figure 6) as well as nAb titers (Figure 3). Although we did not evaluate two-immunization regimens for side-by-side comparison, we highly suspect that nAbs elicited after three immunizations would be more durable than those induced after only two immunizations. In this regard, while our RBD immunogen can be used as a standalone vaccine candidate, it could also be used as a boosting antigen for other vaccine candidates, especially the viral vector vaccines that usually cannot be used more than twice due to immune responses elicited against viral vectors.

A surprising finding from our epitope characterization data was the differences in the linear immunogenic epitopes between the Zn-chitosan and Aluminum-based adjuvant groups (Figures 7, 8). While antibodies induced in the Zn-chitosan group were able to bind to many peptides, those induced in Alhydrogel or Adju-Phos groups only recognized two. This could be due to differences in how antigens are processed by antigen presenting cells and/or how they are presented to B cells. We are not sure at this time whether this is beneficial for eliciting nAbs or not. Although nAbs were induced in the Zn-chitosan group faster, it is possible that induction of nAbs may have nothing to do with eliciting antibodies that could bind peptides. In any event, additional immunological and structural studies at the monoclonal level are needed to better characterize this phenomenon.

In conclusion, results of our study clearly demonstrate that the RBD of S protein is sufficient to elicit potent nAbs against SARS-CoV-2 and that it is a highly promising vaccine candidate.

## Materials and Methods

### Cloning, Expression, and Purification of RBD Immunogen

To generate our RBD-based vaccine candidate, we used the sequence of Wuhan-Hu-1 strain of SARS-CoV-2 (GenBank: MN908947.3). A DNA fragment encoding the following elements were synthesized by Twist Biosciences and cloned into pTwist-CMV-Hygro mammalian expression vector to generate pCOVID-19-RBD: (1) A μ-phosphatase secretory signal peptide (MGILPSPGMPALLSLVSLLSVLLMGCVAE), (2) N-terminal flanking six amino acids (KLTGGT), (3) RBD (from T^333^ to K^528^ of the S protein), (4) C-terminal flanking four residues (GPGM) followed by a six-Histidine tag. The additional amino acid residues flanking the N- and C-terminal ends of the RBD were added for restriction sites (*Hind* III/*Age* I/*Kpn* I, and *Xma* I/*Nsi* I, respectively) to facilitate transfer of the gene into different expression vectors. Following cleavage of the signal peptide, the C-terminal E residue is expected to remain on the final antigen. Additional non-coding sequences were used at the ends of the gene to ensure optimal Kozak sequence and compatibility with the plasmid vector. The final construct was sequenced to confirm intactness of the open reading frame.

The plasmid was transfected into Freestyle 293F cells (Invitrogen) using 293Fectin (Gibco) according to the manufacturer's instructions. Briefly, 2 μl of 293Fectin was mixed with 1μg of DNA and added to 293F cells at a final density of 1 × 10^6^/ml. 293F cells were cultured in FreeStyle^TM^ 293 expression medium (Gibco) in suspension at 37°C under 8% CO_2_ with shaking at 150 rpm. Cell culture medium was harvested 5 days after transfection and the protein was purified by affinity chromatography using Ni-NTA resin (Qiagen). Briefly, cell culture medium, clarified by centrifugation at 2,500 × g for 30 min, was loaded into the Ni-NTA column and washed with washing buffer (50 mM Na_2_HPO_4_, 300 mM NaCl, 20 mM imidazole, pH 8.0), and eluted with elution buffer (washing buffer containing 250 mM imidazole). The eluted fractions were concentrated using Amicon Ultra concentrator (Millipore) with a 10 kDa cut-off filter. Protein concentration was determined by measuring absorbance at 280 nm using multi-mode microplate reader (BioTek Synergy **2**). Protein purity and integrity were analyzed by sodium dodecyl sulfate polyacrylamide gel electrophoresis (SDS-PAGE) followed by visualization of the protein using PAGE Blue stain (Invitrogen).

### CR3022 Expression and Purification

A plasmid construct that encodes heavy and light chain genes of a monoclonal antibody (mAb) CR3022 in backbone of pDR12, a two-promoter plasmid, was kindly provided by Dr. Tianlei Ying (Fudan University, Shanghai, China). CR3022 was expressed by transfecting the plasmid into FreeStyle 293F cells with 293Fectin transfection reagent. Cell culture medium was harvested 5 days after transfection and clarified by centrifugation. Clarified medium was diluted 1:1 with protein A IgG Binding Buffer (Thermo Scientific) and filtered through 0.22 μm filter to remove any precipitate before incubation with Protein A Plus Agarose (Thermo Scientific). After binding, antibody was eluted from the column using low pH IgG Elution Buffer (Thermo Scientific) and neutralized immediately in collection tubes. The purified antibody was buffer-exchanged with PBS and concentrated using Amicon Ultra concentrator with a 30 kDa cut-off filter.

### Characterization of RBD Glycosylation and Binding to CR3022

Purified RBD was deglycosylated using endoglycosidase H (Endo H) or peptide-*N*-glycosidase F (PNGase F) according to the manufacturer's protocol (New England Biolabs). Briefly, 5 μg of RBD was denatured for 10 min at 100°C, and either 10 U of Endo H or PNGase F was added. The reaction mixture was incubated at 37°C for 2 h and then analyzed by SDS-PAGE.

Binding of RBD to CR3022 was tested by standard ELISA. Each well of MaxiSorp plates (Nunc) was coated with 100 ng of RBD in 100 μl coating buffer (0.1 M Na_2_CO_3_/NaHCO_3_, pH 9.6) overnight at 4°C, followed by blocking with 5% calf serum (CS) and 2.5% skim milk in PBS. Then, 3-fold serially diluted CR3022 was added at indicated concentrations and incubated for 2 h at 37°C. After washing (PBS, 0.05% Tween 20), goat anti-human IgG antibody conjugated with horseradish peroxidase (HRP, SouthernBiotech) was added and incubated for 1 h at 37°C. After another wash, TMB substrate (3,3′,5,5′-Tetramethylbenzidine, BioRad) was added and the reaction was stopped by adding 2N H_2_SO_4_. Absorbance at 450 nm (A450) was measured using Spectra Max microplate reader (Molecular Devices Inc.).

### Mice Immunization

Five to six weeks old female BALB/c mice were purchased from the Jackson Laboratory (Bar Harbor, ME). Mice were group housed in a temperature-controlled environment at 22–24°C with 12 h day-night cycles and received food and water *ad libitum*. All animal experiments were performed in Laboratory Animal Resource (LAR) facility of College of Veterinary Medicine, Iowa State University, in accordance with approved protocol (IACUC-20-018) and guidelines of Institutional Animal Care and Use Committee (IACUC).

Mice (4 or 5 per group) were primed intraperitoneally with 30 μg of RBD with or without adjuvants in 200 μl volume. Mice were subsequently boosted twice with 20 μg of RBD 2 and about 5 weeks after the first immunization. Alhydrogel or Adju-Phos (Invivogen) were mixed 1:1 (v/v) with RBD as per manufacturer's recommendation. Zn-chitosan was prepared (19, 57) and mixed with RBD (1,000:1, w/w) for 3 h prior to immunization. Mock controls were immunized with PBS only. Mice were bled from saphenous vein for serum collection prior to first immunization (pre-immune) or after immunization at indicated times. For this initial study, the primary objective was to compare each vaccine group with the mock vaccinated group (PBS), not between the vaccine groups, with respect to their ability to induce antigen-binding or neutralizing antibodies. For this type of analyses in inbred mice, a sample size of 4 in each group is sufficient and will have >95% power to detect an effect size of ≥3.1 with statistical significance of 0.05.

### ELISA for Characterizing Antibody Responses

RBD-specific antibody titers in serum samples collected at different times after immunization were determined by standard ELISA as described above. Wells were coated with 100 ng of the same RBD antigen used for immunization. After blocking, serum samples were used at indicated dilutions. Due to limited amounts of sera, most of the analyses were done with pooled samples. Goat anti-mouse IgG conjugated with HRP (SouthernBiotech) was used as a secondary antibody. Assays were done in duplicates. To assess possible antibody responses against the C-terminal 6×His tag on the RBD, we also did ELISA with 100 ng of HIV-1 gp41-28×3 protein (12.4 kD) that also has a C-terminal 6×His tag, which we previously described (22).

To determine immunogenic linear epitopes, ELISA was done with either a panel of overlapping 17-mer peptides obtained from BEI Resources (NR-52402: NIAID, NIH) or biotinylated peptides (P1–P9, provided by NeoVaxSyn, Inc, synthesized by Synpeptide, Shanghi, China). Peptides were solubilized in PBS (50% DMSO). 17-mer peptides were coated onto ELISA plates overnight at 4°C using a standard coating buffer (200 ng/well). For biotinylated peptides, streptavidin (Thermo Scientific) was first coated onto ELISA plates overnight (300 ng/well). After blocking with 1% BSA, 100 ng of biotinylated peptides were allowed to bind streptavidin for 1 h. For both sets of peptides, 1:300 diluted serum samples were used. The rest of the assays were done as described above.

To compare antibody avidity, ELISA was done using indicated serum samples in the absence or the presence of sodium thiocyanate (NaSCN, BeanTown Chemical, Hudson, NH). Briefly, after binding primary antibodies (1:1,800 diluted sera), plates were washed and the wells were incubated with 100 μl of 0, 1, 2, or 3 M NaSCN in PBS for 15 min. Subsequently, solutions were removed, and the rest of the assay was done as descried above. Relative avidity index was calculated as a percentage of absorbance in NaSCN-treated wells compared to untreated wells.

### SARS-CoV-2 Microneutralization (MN) Assay

SARS CoV-2 (ATCC CRL-1586) isolated from a COVID-19 patient in Washington, USA, was acquired from ATCC. Confluent monolayers of VeroE6 cells (CRL-1586; ATCC) were infected and cultured in Dulbecco's Modified Eagle Medium (DMEM, Corning) containing 5% FBS at 37°C under 5% CO_2_. Virus was passaged three times to generate a stock. Virus titer was determined to be 2.5 × 10^6^ PFU/ml by plaque-forming assay. The stock was aliquoted in small volumes and stored at −80°C until use. All work with infectious SARS-CoV-2 were done in a BSL3 facility at Iowa State University and approved by the Institutional Biosafety Committee (IBC protocol # 20-073).

Vero E6 cells were plated in flat bottom 96-well-plates at a density of 2 × 10^4^ cells/well and incubated overnight at 37°C at 5% CO_2_. Pooled or individual serum samples were diluted with DMEM (1:15) and heat inactivated at 56°C for 30 min. For the MN assay, 50 μl of 3-fold serial dilutions of the sera were prepared in triplicate, starting at 1:15 or 1:45, and incubated with equal volume of 50 PFU SARS-CoV-2 virus (for final dilutions of 1:30 or 1:90, respectively) for 1 h at 37°C. Serum/virus mixtures were transferred into the wells of 96-well-plates with confluent monolayers of VeroE6 cells. After incubation for 1 h at 37°C, supernatant was removed, and 200 μl of fresh DMEM containing 5% FBS was added. Plates were incubated for 3 days at 37°C, 5% CO_2_. Subsequently, cell culture medium from each well was collected for downstream assays.

To assess neutralization activity, we measured lactate dehydrogenase (LDH) activity in cell culture medium using CyQuant LDH Cytotoxicity assay kit (Invitrogen) as per the manufacturer's recommended protocol. LDH is an enzyme found in the cytoplasm, which is released into culture medium when cells lyse. Thus, LDH activity is directly proportional to number of cells lysed upon virus infection. Briefly, 50 μl of cell culture medium was transferred to a 96-well flat bottom plate and mixed with 50 μl reaction buffer. The plate was incubated at room temperature for 30 min. Absorbance (A) was measured at 490 and 680 nm and corrected absorbance was obtained by subtracting 680 nm absorbance from 490 nm absorbance. LDH activity in uninfected and infected cells were used as negative and positive controls, respectively. Infectivity was calculated as: (*A*_virus/serum_ – *A*_uninfected_)/(*A*_virus_ – *A*_uninfected_). Neutralization titers (e.g., NT_50_ or NT_80_) represent reciprocal of the highest serum dilutions that result in 50 or 80% protection, respectively.

To validate LDH-based assays, we also conducted RT-qPCR assays using the culture media of cells from the MN assay. Briefly, 100 μl of cell culture media were collected and mixed with 400 μl of trizol. 80 μl of chloroform was added and centrifuged at 12,000 × g at 4°C for 15 min. Two hundred microliters of isopropanol was added to the upper aqueous layer and centrifuged for 10 min at 12,000 × g and 4°C. RNA pellet was washed with ethanol and resuspended in 50 μl of water. RT-qPCR of the extracted RNA was performed by using Luna Universal Probe One-Step RT-qPCR kit and primer-probe from IDT 2019-nCoV kit on the Applied Biosystems QuantStudio 3 real-time PCR system.

### Structural Analyses

Visualization and analyses of RBD structures (PDB: 6M0J) were done using UCSF Chimera (58). To generate footprints of nAbs, a tool in Chimera (Clashes/Contacts) was used to identify residues on RBD that contact nAbs. Default contact criteria of VDW overlap ≥ −0.4 Å was used.

## Data Availability Statement

The raw data supporting the conclusions of this article will be made available by the authors, without undue reservation.

## Ethics Statement

The animal study was reviewed and approved by Iowa State University IACUC.

## Author Contributions

MC: designed the RBD immunogen, supervised all experiments, analyzed all data and interpreted results, generated final figures, and wrote the manuscript. VS: expressed and purified immunogen, conducted animal experiments, carried out ELISA-based assays, participated in virus neutralization assays, participated in analyzing data, and assisted writing the manuscript. LN: conducted animal experiments, characterized RBD antigenicity, and assisted writing the manuscript. KP: conducted virus neutralization assays, assisted writing the manuscript. BB: analyzed neutralization assay data, assisted writing the manuscript. All authors contributed to the article and approved the submitted version.

## Conflict of Interest

MC has an equity interest in NeoVaxSyn Inc. and serves as the CEO/President. Iowa State University has filed a provisional patent application for the use of RBD as a potential COVID-19 vaccine candidate and will be submitting a final patent application. The remaining authors declare that the research was conducted in the absence of any commercial or financial relationships that could be construed as a potential conflict of interest.
